# Using IPA tools to characterize molecular pathways underlying the involvement of IRF7 in antiviral response to HIV

**DOI:** 10.1515/nipt-2022-0009

**Published:** 2022-03-25

**Authors:** Nikhil K. Kota, Michael Vigorito, Velu Krishnan, Sulie L. Chang

**Affiliations:** Institute of NeuroImmune Pharmacology, Seton Hall University, South Orange, NJ, USA; Department of Biological Sciences, Seton Hall University, South Orange, NJ, USA; Department of Psychology, Seton Hall University, South Orange, NJ, USA

**Keywords:** bioinformatics, gene knockout, HIV, ingenuity pathway analysis, IRF7, neuronal survival

## Abstract

**Objectives:**

Interferon Regulatory Factors (IRFs) regulate transcription of type-I interferons (IFNs) and IFN-stimulated genes. We previously reported that IFN-regulatory factor 7 (IRF7) is significantly upregulated in the brain of HIV-1 transgenic (HIV-1Tg) rats compared to F344 control rats in a region dependent manner [Li MD, Cao J, Wang S, Wang J, Sarkar S, Vigorito M, et al. Transcriptome sequencing of gene expression in the brain of the HIV-1 transgenic rat. PLoS One 2013]. The RNA deep-sequencing data were deposited in the NCBI SRA database with Gene Expression Omnibus (GEO) number GSE47474. Our current study utilized QIAGEN CLC Genomics Workbench and Ingenuity Pathway Analysis (IPA) to identify molecular pathways underlying the involvement of IRF7 in the HIV antiviral response.

**Methods:**

The differential RNA expression data between HIV-1Tg and F344 rats as well as HAND+ and HIV+ cognitively normal patients was collected from GSE47474 and GSE152416, respectively. The “Core Expression Data Analysis” function identified the significant canonical pathways in the datasets with or without IRF7 and its 455 associated molecules.

**Results:**

It was found that IRF7 and its 455 associated molecules altered the expression of pathways involving neurotransmission, neuronal survival, and immune function.

**Conclusions:**

This *in-silico* study reveals that IRF7 is involved in the promotion of macrophage activity, neuronal differentiation, the modulation of the Th-1/Th-2 ratio, and the suppression of HIV-1 translation. Furthermore, we demonstrate that bioinformatics tools such as IPA can be employed to simulate the complete knockout of a target molecule such as IRF7 to study its involvement in biological pathways.

## Background

IRF7, also known as Interferon Regulatory Factor 7, is a well characterized transcription factor that is known to regulate type I interferons (IFNs) against pathogenic infections from parasites, fungi, bacteria, and viruses. This antimicrobial and antiviral response begins with the recognition of microbial products and DNA by pathogen recognition receptors (PRRs) and Toll-like receptors (TLRs) [1]. These signals transduce molecules of the IFN-regulatory factor family (IRF), such as IRF3 and IRF7, in order to activate genes encoding IFN alpha/beta [2]. These signals lead to IRF7 becoming phosphorylated and translocated into the nucleus from its latent form in the cytoplasm [3]. Additionally, increased evidence suggests the role of the phosphatidylinositol 3 kinase pathway in the activation of IRFs, including IRF7 [4]. The induction of high levels of IFN alpha/beta regulate the immune response and their levels are dramatically increased due to the positive feedback loop created by IRF7 [5].

One of the most prevalent pathogenic viral infections worldwide is caused by Human Immunodeficiency Virus (HIV). According to current data, more than 75 million people around the world have been infected with HIV and 38 million people are currently living with the infection [6]. HIV is a retrovirus with the ability to integrate its DNA into the host genome after viral entry [7]. The virus is characterized by causing the cellular death of CD4+ Helper T-cell and modulating the ratio of CD4+/CD8+ cells in patients. It primarily gains entry through CD4 receptors on T lymphocytes, although it is also able to co-infect dendritic cells [8]. Additionally, research has shown that HIV can establish latent infection within memory CD4+ T cells that enter long periods of proliferation and renewal [9].

The immune response to HIV infection begins with PRR’s recognition of pathogen associated molecular patterns (PAMPs) in CD4+ cells [9]. Viral transcriptase products are sensed by intracellular PRRs including interferon inducible protein 16 (IFI16) and cyclic GMP–AMP synthase (cGAS) [10]. As previously stated, the activation of PRRs leads to signaling of IRFs, including IRF7, that induce the expression of type 1 IFN genes that effect many antiviral actions. Host intrinsic restriction factors are expressed that limit HIV replication and spread. These include proteins such as APOBEC3, TRIM5a, SAMHD1, tetherin, SLFN11, IFITM and MX2 [11]. These proteins express their viral restriction activity in various ways. SLFN11 binds to tRNAs and modulates their availability for HIV protein synthesis, while IFITM block HIV entry by colocalizing with HIV viral proteins Env and Gag and MX2 blocks the viral uncoating process to prevent viral integration into the host genome [12, 13].

HIV-1 transgenic (HIV-1Tg) rats appear to have significant behavioral deficits, as indicated in their decreased performance in the Morris Water Maze [14, 15]. RNA sequencing analysis of the HIV-1Tg rat brain found that IRF7 is significantly upregulated in the prefrontal cortex, striatum, and hippocampus compared to the control Fischer 344 (F344) rat [16]. The resulting abnormal gene expression suggested that IRF7 may be involved in deficits relating to learning, memory, and vulnerability to drug addiction observed in the HIV-1Tg rat model [17, 18] and HIV-positive individuals with diagnoses of HIV-Associated Neurocognitive Disorders (HANDs) [19]. The deep RNA-sequencing data were deposited in the NCBI SRA database with Gene Expression Omnibus (GEO) number GSE47474. Another study simulating the partial knockout of IRF7 via CRISPR/Cas9 gene editing in the human embryonic 293FT cell line found that IRF7 is involved in nicotine’s attenuation of the innate antiviral immune response following poly I:C stimulation, a synthetic double stranded RNA analogue that induces an innate immune response [20].

**Figure 1:**
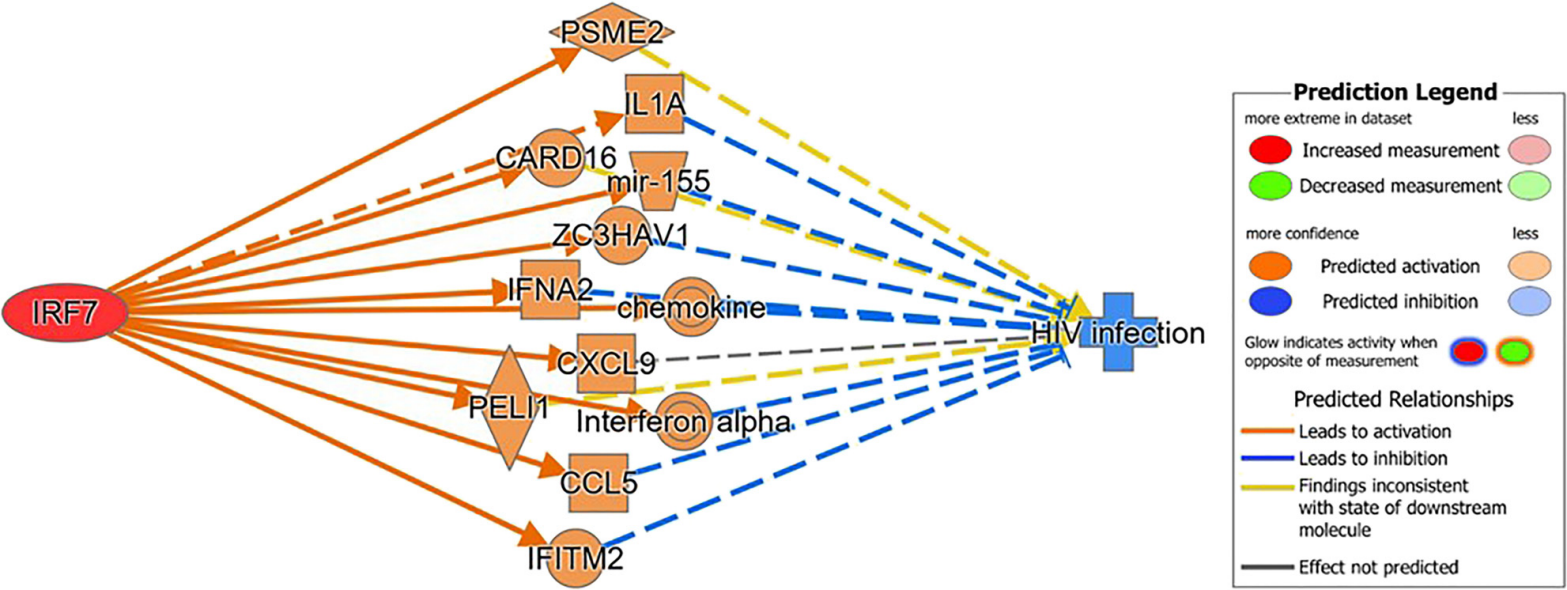
The molecules associated with IRF7 and HIV-infection pathology were identified in reference to the QKB. The overlapping molecules between these two sets of associated molecules were found. The simulation of the upregulation of IRF7 was shown to inhibit HIV-infection pathology through their overlapping associated molecules, demonstrating that IRF7 could play an antiviral role against HIV.

Given the previous literature on IRF7, this study seeks to elucidate what role IRF7 plays in antiviral response to HIV. This study utilized QIAGEN’s CLC Genomics workbench to collect the differential expression between F344 rats and HIV-1Tg rats from the genomics data deposited in the GSE47474 dataset. Likewise, the differential expression between control patients and individuals positive for HIV was collected using the genomics data deposit in the GSE152461 dataset [19]. QIAGEN’s Ingenuity Pathway Analysis (IPA) was used for data mining and the generation of connectivity mapping between IRF7 and HIV-infection and Neuronal Cell Death based on the manually curated publications stored in the QIAGEN Knowledge Base (QKB). In reference to the QKB, the molecules associated with IRF7 and with HIV-1 infection pathology were identified. Figure 1 displays the overlapping molecules associated with IRF7 and HIV-infection pathology. Using Molecular Activity Predictor (MAP) tool to simulate upregulation of IRF7 leading to inhibition of HIV-infection pathology through their overlapping associated molecules, This demonstrated IRF7's possible antiviral role toward HIV infection. With this premise, the molecules associated with IRF7 were removed from the molecular datasets (GSE47474 and GSE152416) to simulate the complete knockout of IRF7. Canonical analyses with and without IRF7 and its associated molecules were conducted and compared to determine the changes brought upon by IRF7 and its associated molecules in biological systems.

## Methods and materials

### CLC Genomics Workbench

The CLC Genomics Workbench CL license was purchased from QIAGEN for the use of all features and tools in the CLC Genomics Workbench (version 22). CLC Genomics Workbench is a bioinformatics tool used to analyze, compare, and visualize genomics data. In this study, the “SRA Search” tool was used to download the GSE 47474 dataset (accession ID: SRR869044), comprising of RNA sequencing data from the prefrontal cortex (PFC), striatum (ST), and hippocampus (HIPP) of F344 Saline and HIV1-Tg transgenic rats, and the GSE152461 dataset (accession ID: SRR854758), comprising of RNA sequencing data from the prefrontal cortex of patients with HIV. Using the “Differential Expression” tool, a statistical differential expression test was run between the experimental (HIV-1Tg rat) and control (F344 rat) samples in the GSE47474 dataset, separated by region (PFC, ST, and HIPP) to identify the gene expression changes in the respective regions. Likewise, a second statistical differential expression test was run between the experimental (HIV + HAND) and control (HIV + cognitively normal) samples from the GSE152461 dataset. The significant gene expression changes for p<0.05 and p<0.005) were identified and uploaded to IPA for further analysis.

### Ingenuity Pathway Analysis Software

**Figure 2:**
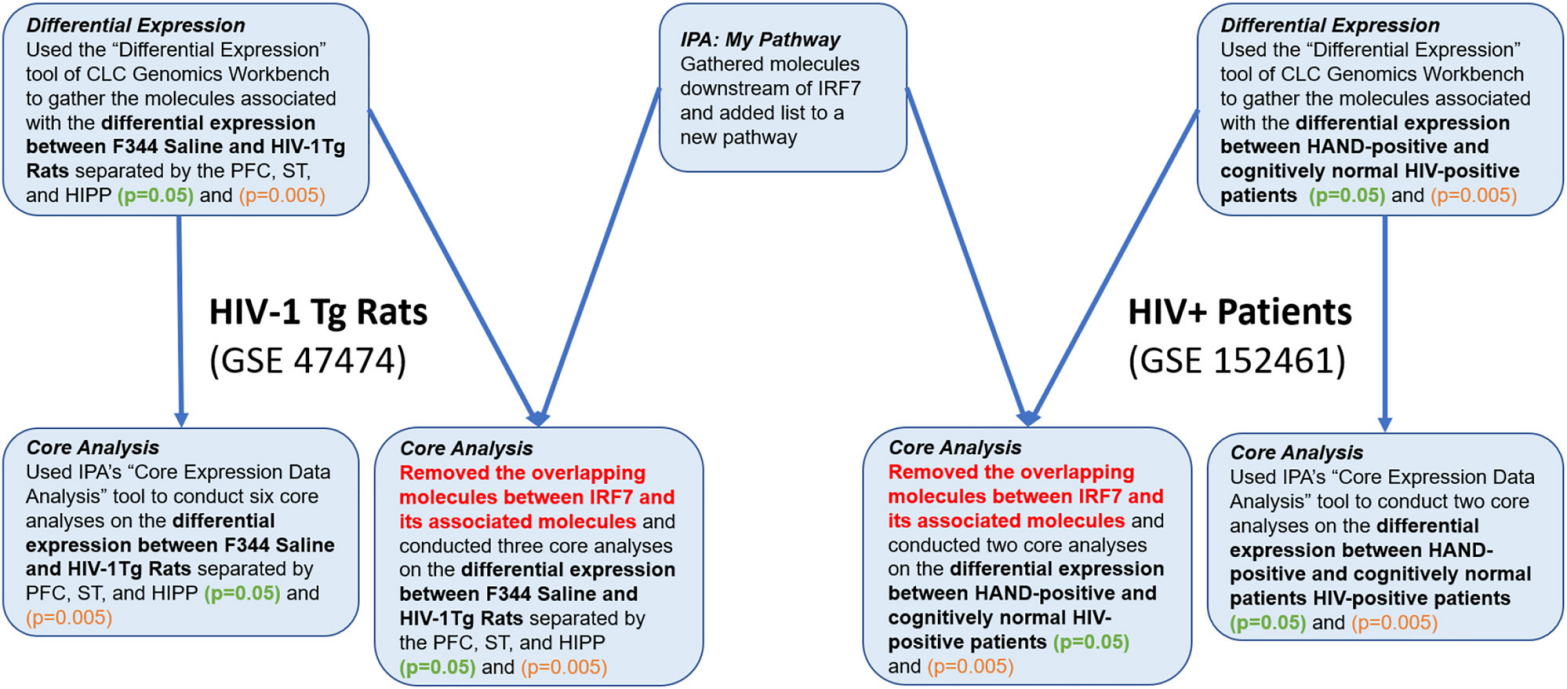
The flow of steps involved in using the bioinformatics and data collection tools offered by QIAGEN to simulate the complete knockout of IRF7 and its associated molecules in F344 rats and HIV-positive individuals. First, the “Differential Expression” tool of CLC genomics workbench was used to determine the differential expression between F344 Saline and HIV-1Tg rats and between HAND-positive and cognitively normal HIV-positive individuals. Then, the a “Canonical Pathway Analysis” was performed on the molecular datasets. Next, the “My Pathway” tool of IPA was used to identify the 455 molecules associated with IRF7. The overlapping molecules between the molecules associated with IRF7 and the molecules in the differential expression datasets were removed. Finally, the “Canonical Pathway Analysis” was performed on the datasets without the 455 molecules associated with IRF7.

The IPA Analysis Match CL license was purchased from QIAGEN for the use of all features and tools in the IPA software (QIAGEN Inc., https://www.qiagenbioinformatics.com/products). IPA is a bioinformatics tool used to analyze data and biological processes using data from the QKB. The flow of steps used to identify and simulate the manipulation of IRF7-related genes and molecules is outlined in Figure 2. The genes involved in the GSE47474 dataset and GSE152461 were uploaded to IPA to be used in conjunction with data from the QKB on IRF7. The “Core Expression Data Analysis” feature of IPA was used to analyze the molecular datasets. The “Core Analysis” provides a “Canonical Pathway Analysis”, an “Upstream Analysis”, and a list of significant “Disease and Functions”. The “Canonical Pathway Analysis” reveals the canonical pathways within the molecular dataset that are statistically predicted to be involved using a −log(p value) calculated by the Benjamini–Hochberg Corrected Fisher’s Exact Test. Core analyses were run on the differential expression datasets of each brain region (prefrontal cortex, striatum, and hippocampus) with a p-value of 0.05 and 0.005. The “My Pathway” tool was used to identify the molecules associated with IRF7 from the QKB. After removing IRF7 and its associated molecules from each of the datasets, an additional six core analyses were run. Using the “My Pathway” tool of IPA, IRF7, CREBBP, HIV-Infection, and Neuronal Cell Death were added to a pathway. Next, the “Path Explorer” tool of IPA was used to connect IRF7 to CREBBP and CREBBP to HIV-Infection and Neuronal Cell Death. Finally, the Molecular Activity Predictor (MAP) tool was used to simulate the increased expression of IRF7 and determine its effects on the expression of CREBBP, HIV-Infection, and Neuronal Cell Death nodes. All data used for this study were retrieved from the QKB between December 23, 2021 until February 23, 2022.

**Figure 3:**
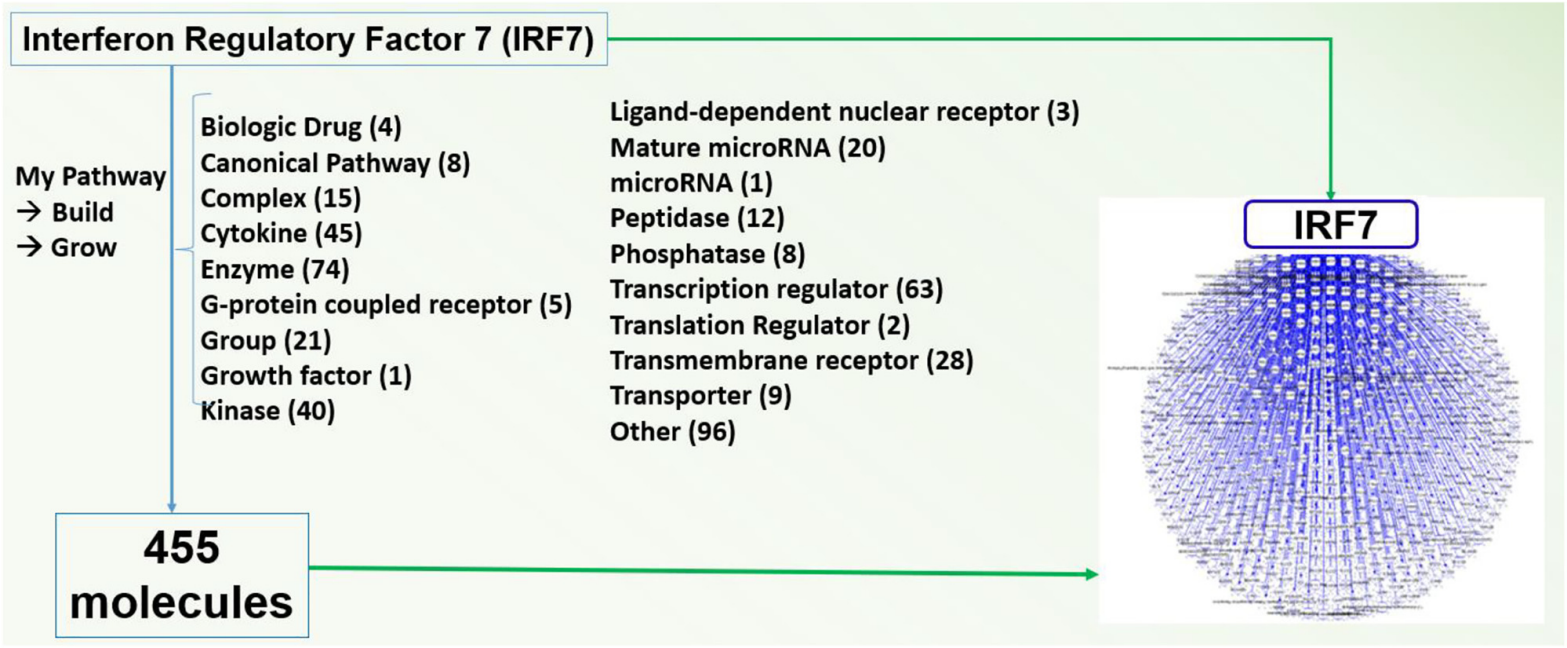
Using the QKB and the build tool of IPA, 511 molecules were found to be associated with IRF7. The above figure lists the 455 molecules of the 511 molecules that naturally occur in biological systems.

## Results

### Overlap between molecules associated with IRF7 and the molecules associated with the differential expression between F344 Saline Rats and the HIV-1 TG rats in the GSE47474 dataset and the GSE152461 dataset.

**Figure 4:**
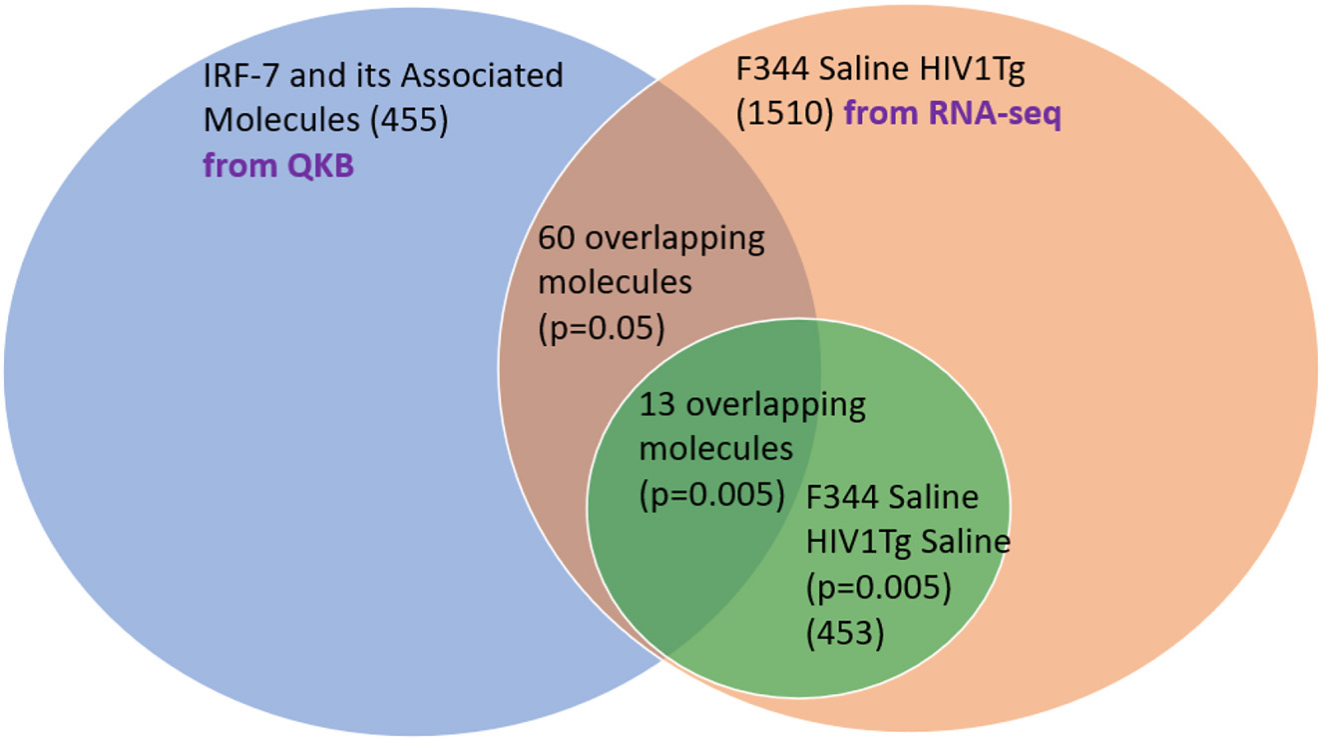
Overlap of the biologically relevant molecules associated with IRF7 with the molecules in the differential expression between F344 and HIV-1Tg rats.

**Figure 5:**
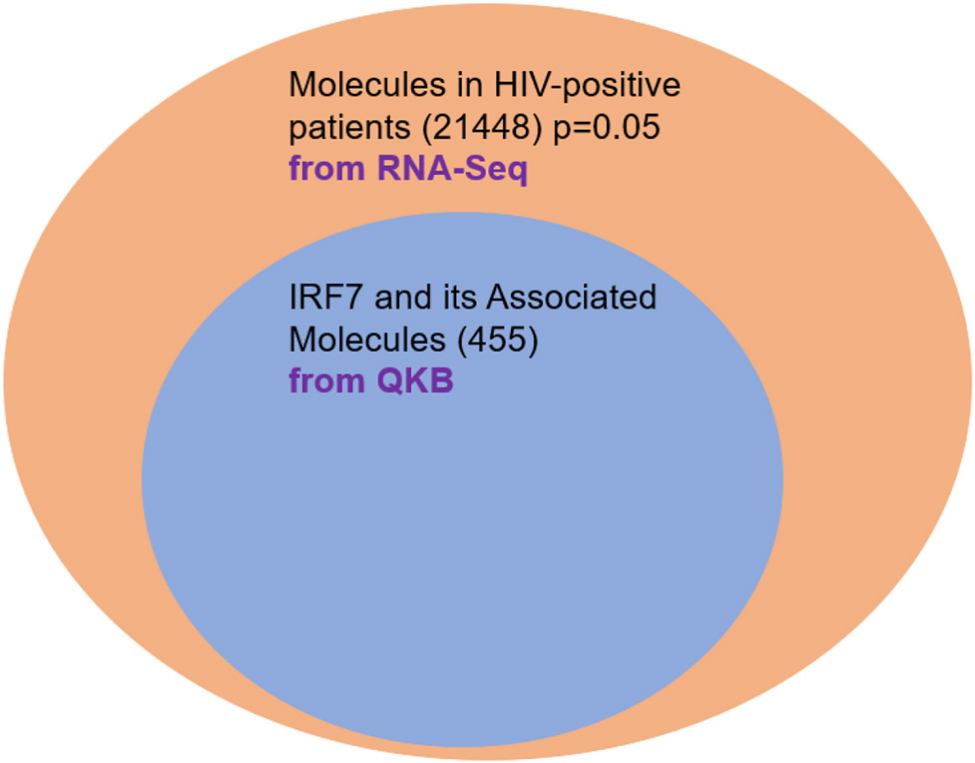
Overlap of the biologically relevant molecules associated with IRF7 with the molecules in the differential expression between HAND-positive and cognitively normal HIV-positive patients.

Using IPA’s “Build” tool to create a custom pathway, 511 molecules were associated with IRF7 (Figure 3). Of these 511 molecules, 56 molecules were removed for not naturally occurring in biological systems (i.e., chemical drugs and toxicants). Of the remaining 455 molecules associated with IRF7, 60 molecules were found to be overlapping between the GSE47474 dataset (Figure 4 and Table 1). However, within each of the three regions of the brain samples collected in the GSE47474 dataset, there were differences in the number of overlapping molecules. The number of overlapping molecules between the molecules associated with IRF7 and the molecules in the three different brain regions (PFC, ST, HP) in the GSE47474 dataset was 42, 22, and 18, respectively. All of the 455 molecules associated with IRF7 were found to be overlapping with the GSE152461 dataset (Figure 5).

**Table 1:** Overlapping Molecules between the GSE47474 dataset and Molecules Associated with IRF7.

Molecule	Prefrontal cortex	Striatum	Hippocampus	Entrez gene name	Molecular family
ACADS	✓	✓		Acyl-CoA dehydrogenase short chain	Enzyme
ACKR2	✓			Atypical chemokine receptor 2	G-protein coupled receptor
ADAR	✓			Adenosine deaminase RNA specific	Enzyme
Ccl2	✓			Chemokine (C-C motif) ligand 2	Cytokine
CD69^a^	✓			CD69 molecule	Transmembrane receptor
CREBBP^a^	✓			CREB binding protein	Transcription regulator
CSF1	✓			Colony stimulating factor 1	Cytokine
CXCL10^a^	✓			C-X-C motif chemokine ligand 10	Cytokine
DHX58		✓	✓	DExH-box helicase 58	Enzyme
DYNC1H1	✓			Dynein cytoplasmic 1 heavy chain 1	Peptidase
EFNA5^a^	✓			Ephrin A5	Kinase
EMX1	✓			Empty spiracles homeobox 1	Transcription regulator
EP300^a^	✓	✓		E1A binding protein p300	Transcription regulator
FLT3	✓			fms related receptor tyrosine kinase 3	Kinase
GBP6	✓			Guanylate binding protein family member 6	Enzyme
HELZ2		✓		Helicase with zinc finger 2	Nucleus
IFI44			✓	Interferon induced protein 44	Other
IFIT3			✓	Interferon induced protein with tetratricopeptide repeats 3	Other
IFNG^a^	✓			Interferon gamma	Cytokine
Igtp			✓	Interferon gamma induced GTPase	Enzyme
IL2RG	✓			Interleukin 2 receptor subunit gamma	Transmembrane receptor
IQSEC1	✓			IQ motif and Sec7 domain ArfGEF 1	Other
IRF7^a^	✓	✓	✓	Interferon regulatory factor 7	Transcription regulator
INSR			✓	Insulin receptor	Kinase
KL		✓		Klotho	Enzyme
Ly6a	✓			Lymphocyte antigen 6 complex, locus A	Other
MAP3K7			✓	Mitogen-activated protein kinase kinase kinase 7	Kinase
MAP3K8^a^	✓			Mitogen-activated protein kinase kinase kinase 8	Kinase
MMP2		✓		Matrix metallopeptidase 2	Peptidase
MX1	✓	✓	✓	MX dynamin-like GTPase 1	Enzyme
Mx2		✓		MX dynamin like GTPase 1	Enzyme
NCOA2^a^			✓	Nuclear receptor coactivator 2	Transcription regulator
NSD2		✓		Nuclear receptor binding SET domain protein 2	Enzyme
OAS1	✓	✓	✓	2′-5′-oligoadenylate synthetase 1	Enzyme
OAS2	✓		✓	2′-5′-oligoadenylate synthetase 2	Enzyme
OASL	✓			2′-5′-oligoadenylate synthetase like	Enzyme
OASI2	✓	✓	✓	2′-5′ oligoadenylate synthetase-like 2	Enzyme
PARP12	✓			poly(ADP-ribose) polymerase family member 12	Enzyme
PARP14	✓	✓	✓	poly(ADP-ribose) polymerase family member 14	Enzyme
PDLIM2		✓		PDZ and LIM domain 2	Other
PGR^a^	✓			Progesterone receptor	Ligand-dependent nuclear receptor
PMAIP1	✓			Phorbol-12-myristate-13-acetate-induced protein 1	Other
PSEN1^a^	✓			Presenilin 1	Peptidase
PSMB10	✓			Proteasome 20S subunit beta 10	Peptidase
PSME1		✓		Proteasome activator subunit 1	Peptidase
PTGER4	✓			Prostaglandin E receptor 4	G-protein coupled receptor
PRL		✓		Prolactin	Cytokine
RSAD2	✓	✓	✓	Radical S-adenosyl methionine domain containing 2	Enzyme
RTP4	✓	✓	✓	Receptor transporter protein 4	Other
SAMSN1	✓			SAM domain, SH3 domain and nuclear localization signals 1	Other
SATB1	✓			SATB homeobox 1	Transcription regulator
SPP1	✓			Secreted phosphoprotein 1	Cytokine
STAT2^a^	✓			Signal transducer and activator of transcription 2	Transcription regulator
STAT6^a^	✓	✓		Signal transducer and activator of transcription 6	Transcription regulator
TLR3		✓		Toll like receptor 3	Transmembrane receptor
TLR5			✓	Toll like receptor 5	Transmembrane receptor
TRAF1	✓			TNF receptor associated factor 1	Other
TUBA1C	✓	✓		Tubulin alpha 1c	Other
UBA7			✓	Ubiquitin like modifier activating enzyme 7	Enzyme
USP18	✓	✓	✓	Ubiquitin specific peptidase 18	Peptidase
✓ Indicates the IRF7-associated molecules identified for each brain region					

^a^Indicates molecules associated with IRF7 with a significance of p=0.005 for every brain region identified.

### Canonical pathways involved in the differential expression with and without the molecules associated IRF7.

The core analyses performed with and without the molecules associated with IRF7 revealed significant changes in several canonical pathways (Table 2). In the GSE47474 dataset, 12, 4, and 4 pathways were found to be significantly impacted (Z-score ≥ +/−2) by the presence of IRF7 and its associated molecules in the PFC, ST, and HIPP, respectively. In the GSE152461 dataset, 13 pathways were significantly impacted. Furthermore, some of the significantly impacted pathways in each region and even between the datasets were overlapping. For example, the Estrogen Receptor Signaling pathway increased in the presence of the molecules associated with IRF7 in the PFC and ST of the GSE47474 dataset as well as in the GSE152461 dataset (PFC). The Dopamine-DARPP32 Feedback in cAMP Signaling pathway was found to increase in the PFC of both the GSE47474 and GSE152461 datasets, while the Neuroinflammation signaling was found to decrease in the PFC of both datasets. The Senescence pathway was found to increase in the PFC of the GSE47474 and the GSE152461 dataset. The CREB signaling in neurons pathway was found to increase in the GSE152461 dataset but only in the HIPP of the HIV-1Tg rat dataset.

**Table 2:** Change in Z Score of Significant Canonical pathways in the Presence of IRF7 and its associated molecules.

Canonical pathway	Categorization of canonical pathway	−Log(p-Value)	Change in Z-Score
**2a: Prefrontal cortex**			
Opioid signaling pathway	Neurotransmission	12.8	0.239
Dopamine-DARPP32 feedback in cAMP signaling	Neurotransmission	12.3	0.120
Synaptogenesis signaling pathway	Neuronal survival	10.10	0.202
Calcium signaling	Neurotransmission	9.07	0.256
Estrogen receptor signaling	Neurotransmission	9.93	0.469
Synaptic long-term potentiation	Neuronal survival	9.89	0.322
Senescence pathway	Neuronal survival	8.58	0.268
Neuroinflammation signaling pathway	Neuronal survival	7.45	−0.181
VDR/RXR activation	Neurotransmission	6.31	−0.700
Th1 pathway	Immune	6.45	0.114
Th2 pathway	Immune	6.28	−0.387
Xenobiotic metabolism AHR signaling pathway	Immune	6.10	1.216
**2b: Striatum**			
IL-17 signaling	Immune	8.51	2.449
Xenobiotic metabolism CAR signaling pathway	Immune	8.30	1.342
Pulmonary healing signaling pathway	Neurotransmission	7.31	2.236
Estrogen receptor signaling	Neurotransmission	6.65	2.449
**2c: Hippocampus**			
Role of hypercytokinemia/hyperchemokinemia in the pathogenesis of influenza	Immune	6.646	2.646
Neuroinflammation signaling pathway	Neuronal survival	6.488	2.121
Phagosome formation	Immune	6.177	2.673
CREB signaling in neurons	Neuronal survival	5.147	3.464
**2d: GSE152461 dataset (prefrontal cortex)**			
Dopamine-DARPP32 feedback in cAMP signaling	Neurotransmission	10.89	0.720
CREB signaling in neurons	Neuronal survival	9.24	0.330
Synaptic long term depression	Neuronal survival	9.60	−0.165
Role of PKR in interferon induction and antiviral response	Immune	9.42	0.905
Role of hypercytokinemia/hyperchemokinemia in the pathogenesis of influenza	Immune	8.64	2.646
Adrenomedullin signaling pathway	Immune	7.43	−0.482
Calcium signaling	Neurotransmission	7.28	0.278
Estrogen receptor signaling	Neurotransmission	7.13	0.469
Synaptic long term potentiation	Neuronal survival	6.89	0.322
Senescence pathway	Neuronal survival	6.58	0.268
Neuroinflammation signaling pathway	Neuronal survival	6.34	−0.181
VDR/RXR activation	Neurotransmission	6.31	−0.700
Th1 pathway	Immune	6.45	0.114

Quantitative Expression Changes for the Canonical pathways of IRF7 and Associated Molecules between F344 Saline Rats and the HIV-1 TG Rats in the GSE47474 Dataset and the GSE152461 Dataset.

### IRF7 and its associated molecules

**Figure 6:**
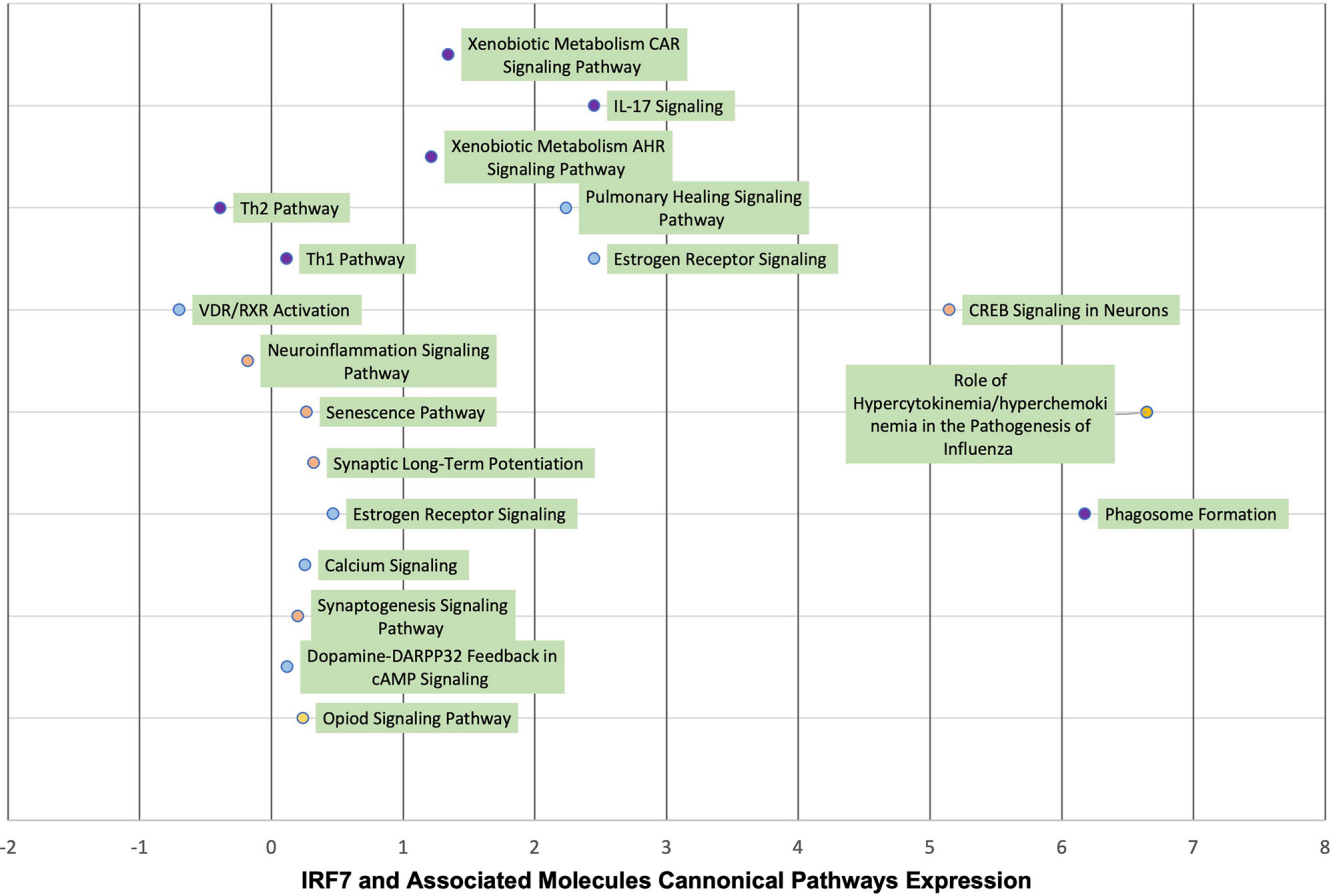
Visual illustration of the quantitative analysis on the relationship between the 60 overlapping molecules between the molecules associated with IRF7 and the differential expression between F344 Saline and HIV-1Tg rats in the GSE47474 dataset. The significant canonical pathways (Z-score >/=+/−2) in the core analyses with IRF7 and its associated molecules were identified, and their corresponding Z-scores were identified. Likewise, the Z-scores of the same canonical pathways in the datasets without IRF7 and its associated molecules were identified. The above figure lists the difference in the Z-scores with and without IRF7 and its associated molecules to determine the changes brought upon by IRF7 and its associated molecules.

**Figure 7:**
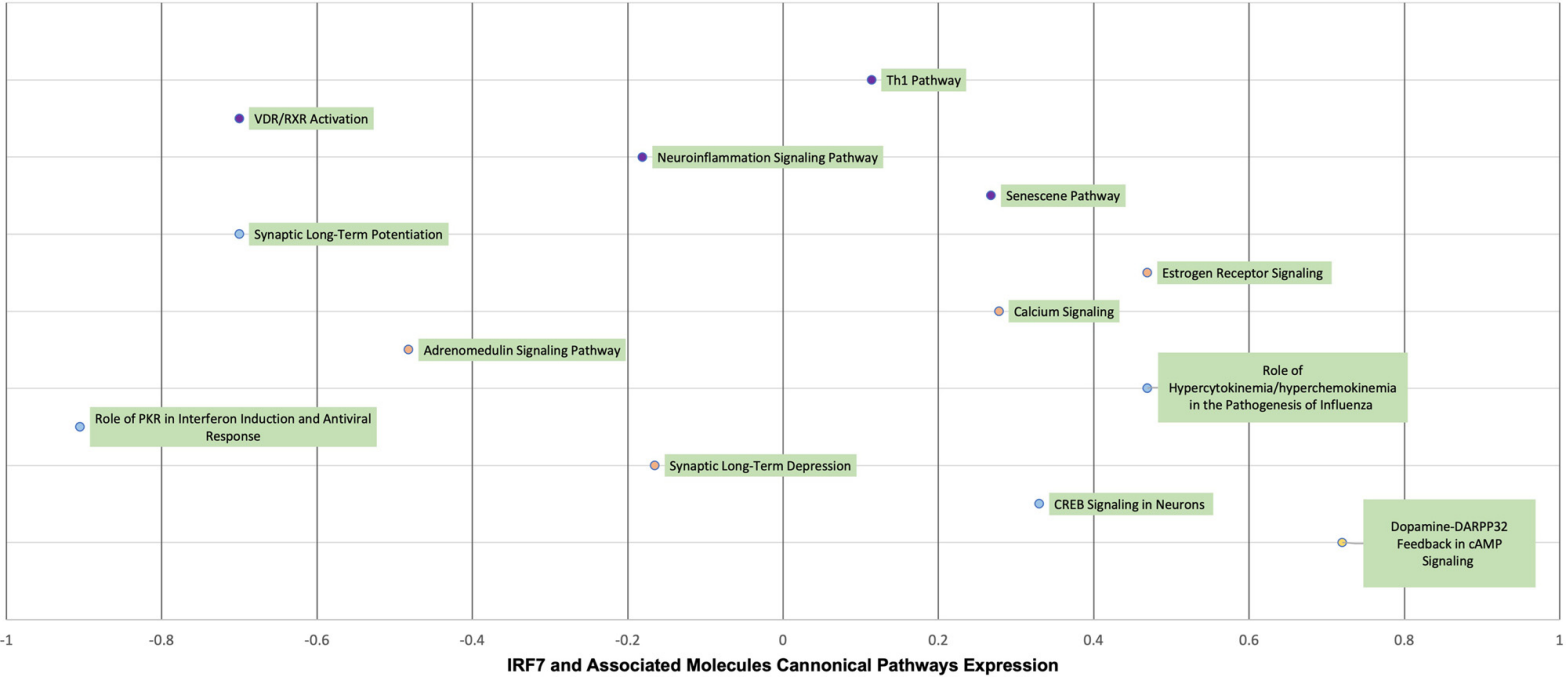
Visual illustration of the quantitative analysis on the relationship between the 455 overlapping molecules between the molecules associated with IRF7 and the differential expression between control patients and HIV-positive patients in the GSE152461 dataset. The significant canonical pathways (Z-score >/=+/−2) in the core analyses with IRF7 and its associated molecules were identified, and their corresponding Z-scores were identified. Likewise, the Z-scores of the same canonical pathways in the datasets without IRF7 and its associated molecules were identified. The above figure lists the difference in the Z-scores with and without IRF7 and its associated molecules to determine the changes brought upon by IRF7 and its associated molecules.

Using the “Core Analysis: Expression Analysis Tool”, IRF7 and its 60 associated molecules were compared to the 705 well-defined canonical pathways within QIAGEN’s Knowledge Base (QKB). The most significant pathways were chosen, which were those that displayed the largest –log(p-value) and demonstrated the strongest overlap between the IRF7 associated molecules. Then, the Z-scores associated with these pathways were found [21, 22]. Additionally, the same “Core Analysis” function was run but without IRF7 and its causally-associated molecules to simulate the knockout of IRF7. Again, the Z-scores associated with these pathways were found. Then these differences in Z-scores (Figures 6 and 7) were calculated to demonstrate the effect that IRF7 and its associated molecules had on pathways related to immune function (purple points), neuronal survival (orange) and neurotransmission (light blue).

The pathways shown in Figure 6 convey Z-score expression changes for the canonical pathways associated with the GSE47474 dataset. The majority of the pathways experienced increased expression or upregulation while 3 pathways of note experienced decreased expression or downregulation which were the Th2 pathway (immune), VDR/RXR pathway (neurotransmission), and Neuroinflammation pathway (neuronal survival). Additionally, pathways of note experiencing increased expression were the Th1 pathway (immune), Synaptic Long-term Potentiation (neuronal survival), and CREB signaling in Neurons (neuronal survival).

Similarly, the pathways shown in Figure 7 convey Z-score expression changes for the canonical pathways associated with the GSE152461 dataset. For this dataset, 7 significant canonical pathways experienced increased expression when associated with IRF7 and its molecules while 6 canonical pathways experienced decreased expression. Similar to the GSE47474 dataset, the VDR/RXR pathway and Neuroinflammation pathway experienced decreased expression while the Th1 pathway experienced increased expression. However, while the Synaptic Long-term Potentiation pathway had an expression increase in the GSE47474 dataset, it expressed a decrease in the GSE152461 dataset.

## Discussion

Previously, the deep sequencing of HIV-1Tg rats found that IRF7 was one of the only genes that was significantly upregulated in the prefrontal cortex, striatum, and hippocampus of the HIV-1Tg rats compared to the F344 rats. Given that IRF7 is the master regulator of type I interferon production and aberrant production of type I interferons is associated with autoimmune disorders, IRF7 appeared to be involved in the antiviral response to HIV. In another study, the partial knockout of IRF7 via CRISPR/Cas9 gene editing in the human embryonic kidney 293FT cell line found that in the absence of IRF7, the antiviral response in HIV-1Tg rats decreased [20]. To fully investigate what role IRF7 plays, we simulated the complete knockout of IRF7 and its associated molecules via an *in-silico* approach by using the data compiled in the QKB as well as data retrieved from rat *in vivo* and human *in-vitro* studies. Although taken from different species, the data shows how IRF7 and its associated molecules impact several pathways including neurotransmission-related pathways, immune-related pathways, and neuronal survival related pathways.

### Neurotransmission and neuronal survival-related pathways

**Figure 8:**
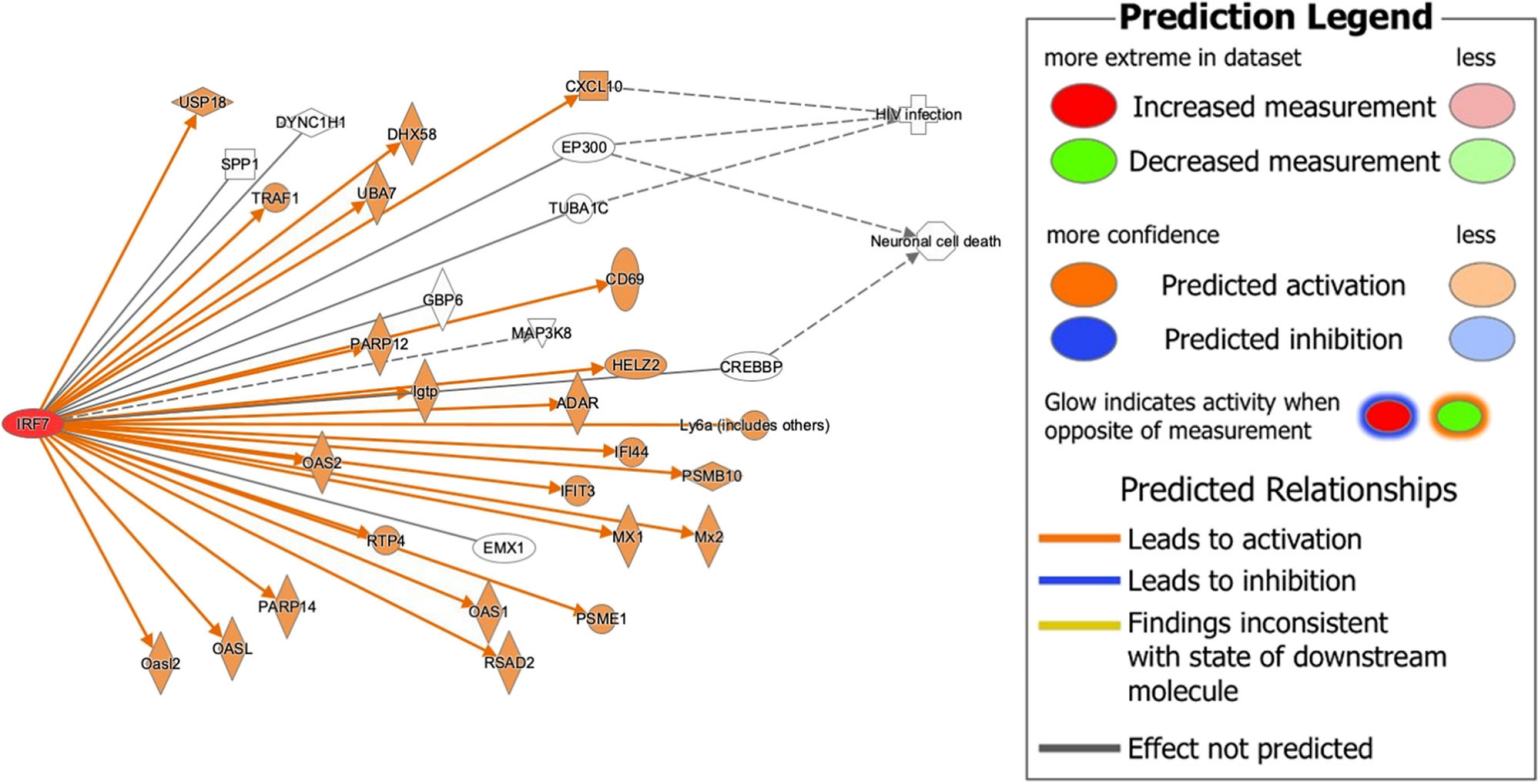
Connectivity map of the relationship between IRF7 and the 13 overlapping molecules between the molecules associated with IRF7 and the molecules in the differential expression between F344 Saline and HIV-1Tg rats in the GSE47474 dataset that influence CREB, neuronal cell death, and HIV infection pathology.

A common pattern in the core analyses of the rat PFC (GSE47474) and the human PFC (GSE152461) dataset in the presence of the molecules associated with IRF7 was the involvement of cAMP Response Element Binding Protein (CREB), which binds to CREB Binding Protein (CREBBP), a molecule downstream of IRF7. The Dopamine-DARPP32 Feedback in cAMP Signaling and the Calcium signaling pathway results in an increase in dopamine and intracellular calcium levels, respectively, both of which lead to increased CREB levels. Increased levels of CREB also appear due to the upregulation of the synaptic long term potentiation pathway by IRF7. In neurons, CREB functions as a transcription factor and binds to cAMP Response Element (CRE), leading to the transcription of several cell survival genes involved in neurogenesis and metabolism [23, 24]. As seen in Figure 8, IRF7 is connected to Neuronal Cell Death via CREBBP. Although IPA did not note a predicted activation or inhibition of CREBBP, it does present a connection between IRF7 and Neuronal Cell Death. Taken together, we find that IRF7 and its associated molecules could possibly reduce neuronal cell death by increasing the expression of CREB via the CREBBP gene.

In addition to increasing CREB, IRF7 and its associated molecules also upregulated the expression of CCL2 in the PFC of F344 rats and in HIV-positive individuals (Table 2). CCL2 is known to induce neuronal differentiation, oligodendrocyte maturation, and myelin production. Low myelin production has been shown to result in neurocognitive changes, even in HIV-positive individuals receiving anti-retroviral therapy [16, 25, 26]. Therefore, the upregulation of CCL2 by IRF7 and its associated molecules may reduce neurodegeneration in the CNS, reducing the cognitive deficits brought upon by HIV-Associated Neurocognitive Disorders (HANDs).

### Immune-related pathways

IRF7 and its associated molecules lead to the activation of canonical pathways involved in the cellular-mediated response to infection. The upregulation of the Th1 pathway in the PFC of HIV-1 Tg rats and HIV-positive individuals with mild neurocognitive deficits (Table 2a and d) leads to the development of T-Helper 1 cells, which aid in the production of IFN-γ, a cytokine that recruits macrophages for the destruction of pathogens and presentation to T-Lymphocytes. The increase in macrophage activity is also reflected in the increase of the phagosome formation pathway (Table 2c). At the same time, there was a decrease in the Th2 pathway in the PFC of F344 rats (Table 2a), leading to the decrease of Th-2 cells, which is involved in the humoral response. A widely accepted hypothesis suggests that the switch from Th1 activity to Th2 activity leads to the progression of Acquired Immunodeficiency Disorder (AIDS) in individuals affected with HIV [27, 28]. Therefore, the simultaneous decrease of Th2 activity and the simultaneous increase of Th1 activity in the PFC suggests that IRF7 and its associated molecules may slow or prevent the development of AIDS in HIV-positive individuals.

The upregulation of the Interleukin-17 signaling pathway was observed in the STR of F344 rats (Table 2b). Given that the low frequency of IL-17-producing cytotoxic T-cells is associated with the persistent immune activation in HIV-positive individuals despite receiving Highly Active Antiretroviral Therapy (HAART) [29], the upregulation of genes in the Interleukin-17 signaling pathway suggests that IRF7 and its associated molecules may prevent chronic immune activation in HIV-positive individuals.

It is hypothesized that HIV-1 translation is modulated by the inducible Protein Kinase RNA-activated (PKR), which phosphorylates the alpha subunit of eukaryotic translation initiation factor 2 (eIF2a). Upon phosphorylation, eIF2a hinders the ternary tRNA^met^-GTP-eIF2 complex, resulting in the formation of stress granules (SGs) which encapsulate viral RNA and transcription and translation-related proteins, thus decreasing viral replication [30, 31]. In this study, we observe the upregulation of the Role of PKR in Interferon Induction and Antiviral Response signaling pathway in HIV-positive individuals (Table 2d), suggesting that IRF7 and its associated molecules may decrease HIV-1 translation by encapsulating the viral RNA and transcription and translation-related proteins needed for viral production.

Using the path explorer tool, a connectivity map between IRF7 and HIV-infection was generated using the data curated from the QKB (Figure 1). The simulated activation of IRF7 lead to the activation of several molecules downstream of IRF7, including several molecules found to be overlapping between the GSE47474 dataset and IRF7 and its associated molecules (Table 1) such as CXCL10 and molecules closely related to the overlapping molecules, such as CXCL9, CXCL8, CD80, CCL8, etc. The activation of said molecules leads to the inhibition of HIV-infection. Although this connectivity map was not generated from the data from the GSE47474 and GSE152461 datasets, it supports our findings that IRF7 can play an antiviral role against HIV-infection via the overlapping molecules between the GSE47474 dataset and IRF7 and its associated molecules.

There are certain limitations to this study that must be considered when interpreting the results. In contrast to the GSE47474 dataset, the GSE152461 dataset used human postmortem tissue samples, which reduces the reliability of the data as humans have much more complex biological systems and are influenced by genetic, environmental, and social factors that are difficult to account for in experimental settings. On the other hand, the F344 and HIV1Tg rats are organisms developed strictly for use in research laboratory settings and thus have less variation in their genome and environment. It is also imperative to note that due to the inherent complexity of biological systems, these core analyses are still isolated networks and do not fully represent how these molecules interact in a biological system. Nevertheless, the results of our *in-silico* study provide an empirically based hypothesis that is testable with *in-vitro* and *in-vivo* studies that can knockout IRF7 and its associated molecules along with the use of bioinformatics tools such as IPA to simulate the complete knockout of a target molecule *in-silico.* These proposed knockout experiments are likely to reveal the role of IRF7 in the antiviral response to HIV, specifically through its involvement in neurotransmission, immune and neuronal survival related pathways.

## Conclusions

Our findings from the complete *in-silico* knockout of IRF7 and its associated molecules provide pertinent information on IRF7’s role in the antiviral response to HIV. By integrating genomics data from the GSE47474 and the GSE152461 datasets, we demonstrate that IRF7 can modulate immune system production, specifically the Th-1/Th-2 ratio, promote neurogenesis through the upregulation of CREBBP and CCL2, and possibly halt the onset of AIDS in HIV-positive individuals.
